# Significant Quantitative and Qualitative Transition in Pituitary Stem / Progenitor Cells Occurs during the Postnatal Development of the Rat Anterior Pituitary

**DOI:** 10.1111/j.1365-2826.2011.02198.x

**Published:** 2011-10

**Authors:** S Yoshida, T Kato, H Yako, T Susa, L-Y Cai, M Osuna, K Inoue, Y Kato

**Affiliations:** * Division of Life Science, Graduate School of Agriculture, Meiji UniversityKanagawa, Japan; † Institute of Reproduction and Endocrinology, Meiji UniversityKanagawa, Japan; ‡ Division of Graduate School of Science and Engineering, Saitama UniversitySaitama, Japan.; § Department of Life Science, School of Agriculture, Meiji UniversityKanagawa, Japan

**Keywords:** prophet of PIT1, pituitary, stem/progenitor cell, SOX2, S100

## Abstract

We reported recently that a pituitary-specific transcription factor PROP1 is present in SOX2-positive cells and disappears at the early stage of the transition from progenitor cell to committed cell during the embryonic development of the rat pituitary. In the present study, we examined the localisation and identification of SOX2-positive and PROP1/SOX2-positive cells in the neonatal and postnatal rat pituitaries by immunohistochemistry. Quantitative analysis of immunoreactive cells demonstrated that SOX2-positive pituitary stem/progenitor cells are not only predominantly localised in the marginal cell layer, but also are scattered in the parenchyma of the adult anterior lobe. In the marginal cell layer, the number of PROP1/SOX2-positive cells significantly decreased after postnatal day 15, indicating that a significant quantitative transition is triggered in the marginal cell layer during the first postnatal growth wave of the anterior pituitary. By contrast, other phenotypes of SOX2-positive stem/progenitor cells that express S100β appeared in the postnatal anterior pituitary. These data suggested that quantitative and qualitative transition occurs by acquisition of a novel mechanism in terminal differentiation in the postnatal development of the anterior pituitary.

The pituitary primordium Rathke's pouch arises from the invaginating oral ectoderm in the early embryonic period and develops into the anterior and intermediate lobes. The anterior lobe is composed of five different endocrine cell types, luteinising hormone (LH)- and follicle-stimulating hormone (FSH)-producing cells (gonadotrophs), thyroid-stimulating hormone (TSH)-producing cells (thyrotrophs), growth hormone (GH)-producing cells (somatotrophs), prolactin (PRL)-producing cells (mammotrophs) and adrenocorticotrophic hormone (ACTH)-producing cells (corticotrophs). Melanotrophs which produce melanocyte-stimulating hormone (α-MSH) are present In the intermediate lobe. In the processes of pituitary organogenesis, these endocrine cells are terminally differentiated under control of numerous signalling factors and transcription factors whose expression and function are coordinated under spatial–temporal regulation. To date, many transcription factors important for pituitary organogenesis, such as HESX1/RPX, PTX1, TBX19, LHX3, LHX4, GATA2, PROP1 (prophet of PIT1), PIT1 and SF1, have been discovered and characterised (1), although their precise functions in pituitary organogenesis are not yet fully understood.

In addition, non-endocrine cells have been found in the pituitary. One of the non-endocrine cells identified so far comprises a type of folliculo-stellate cell, which was first distinguished by electron microscopy (2) and named in light of its characteristic shape and follicle formation capacity (3). Although it was defined later by the presence of S100 protein, its functions in the pituitary and embryonic origin remain obscure (4,5). It is notable that folliculo-stellate cells lining the marginal cell layer facing the residual lumen of the Rathke's pouch have long been hypothesised as pituitary stem/progenitor cells (6–9). Over the last decade, the marginal cell layer has often been proposed to be a pituitary stem/progenitor cell niche providing a cell remodelling system to compensate for pituitary cellular plasticity (10,11). Recent studies have demonstrated that cells expressing the gene for the HMG-box transcription factor SOX2, a marker of stem/progenitor cells, are present in the adult pituitary and have an ability to generate all of the pituitary cell types (12,13). Fauquier *et al.* (12) demonstrated that a small population of progenitor cells, which are present in the adult pituitary gland and express *Sox2*, have an ability to form pituispheres in culture and differentiate into all of the pituitary hormone-producing cell types. Chen *et al.* (13) observed that SOX2 positive cells are more abundant in the pituitary of early-postnatal mice at the age of the first pituitary growth wave (1-week-old) than in adult animals. Thus, SOX2 might have a key role in maintenance of stem/progenitor cells and/or differentiation of pituitary cell lineage. More recently, we presented immunohistochemical observations that a pituitary-specific factor PROP1 consistently coexists with SOX2 throughout the embryonic development of the pituitary (14).

*Prop1* encodes a paired-like homeodomain transcription factor, and is a heritable responsive gene for the combined pituitary hormone deficiency in the *Ames* dwarf mice (*Prop1*-deficient dwarf mice) (15) and human patients showing absence or low levels of GH, PRL, TSH, LH and FSH (16–18), as well as ACTH (19). Longitudinal studies in *Prop1-*deficient dwarf mice from early embryogenesis through adulthood have demonstrated that PROP1 plays roles in morphogenesis, apoptosis and proliferation in the developing pituitary (20), as well as in cell migration in the expanding anterior lobe (21). We further observed that *Prop1*-expression is transient in PIT1-positive cells but absent in endocrine cells during embryonic development, suggesting that PROP1 exists in SOX2-positive stem/progenitor cells and disappears at the early stage of the transition of progenitor cells to committed cells in the embryonic development of the pituitary (14).

In the present study, we aimed to track quantitatively the cells expressing *Prop1* and *Sox2* in the postnatal development of the rat anterior pituitary by the immunohistochemical technique. Finally, we demonstrated that PROP1 is absent in any endocrine cells but consistently coexists with SOX2 in non-endocrine cells, most of which are S100-positive. Analysis of PROP1, SOX2 and S100β-positive cells in the anterior pituitary of S100β-green fluorescent protein (GFP) transgenic rat (22) demonstrated that significant quantitative and qualitative transition in *Sox2*-expressing stem/progenitor cells lining the marginal cell layer occurred in the postnatal development of the anterior pituitary.

## Materials and methods

### Animals

Intact male Wistar-Imamichi strain rats, kept in a controlled environment, were used. S100β-GFP transgenic rat was generated by fusing the *S100β*-promoter to the reporter gene *GFP* (22). The present study was approved by the committee on animal experiments of the School of Agriculture, Meiji University.

### Generation of antibody

**Fig. 1:**
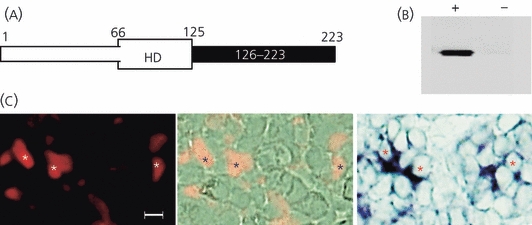
Immunohistochemistry and *in situ* hybridisation (a). Recombinant rat C-terminal region (closed box; amino acid residues 126–223) was used to generate the antibody after purified by sodium dodecyl sulphate-polyacrylamide gel electrophoresis. HD indicates a homeodomain important for DNA binding (amino acid residues 66–125). (b). Western blotting was performed using cell lysate of Chinese hamster ovary cells which overexpressed rat PROP1 cDNA (+) and empty vector (−). (c). Immunohistochemistry with anti-PROP1 antibody (left) and *in situ* hybridisation of *Prop1* mRNA (right) were performed using mirror sections of rat pituitary (P5). Merged image of immunohistochemistry and light microscopy is shown in a center panel. Cells retaining both protein and mRNA signals of *Prop1* are indicated by asterisks (*). Scale bar = 10 μm.

Guinea pig anti-rat PROP1 antiserum was generated as described previously (14). Briefly, the cDNA of rat *Prop1* corresponding to the C-terminal region (amino acid residues 126–223) (Fig. 1a) was cloned into pET32a vector (Novagen, Darmstadt, Germany) to generate the TrxA-His-tag fused protein. After the fusion protein was separated by 12.5% sodium dodecyl sulphate-polyacrylamide gel electrophoresis (SDS-PAGE) and stained with Coomassie Brilliant Blue, the protein band corresponded to fusion protein of the C-terminal region of PROP1 was cut and applied on immunisation in guinea pig.

Verification of the generated anti-PROP1 antibody was accomplished by western blotting first (Fig. 1b) and then by immunohistochemistry (methods described below) and *in situ* hybridisation using mirror sections (8 μm thickness) of male rat pituitary (P5) (Fig. 1c). Western blotting was performed using cell lysate of Chinese hamster ovary cells transfected with expression vector pcDNA3.1 (Invitrogen, Carlsbad, CA, USA) fused with the full length cDNA of rat *Prop1*. After proteins were separated on a 10% SDS-PAGE, electro-blotting to a polyvinylidene fluoride membrane was carried out. The membrane was immunoreacted with anti-PROP1 antibody (dilution 1 : 5000) and then secondary antibody of horseradish peroxidase-conjugated donkey immunoglobulin (Ig)G against guinea pig IgG (dilution 1 : 25 000; Jackson ImmunoResearch, West Grove, PA, USA). Immunoreacted signals were visualised by TMB stabilised substrate for horseradish peroxides (Promega, Madison, WI, USA). *In situ* hybridisation was performed as described previously (23). Briefly, frozen sections were treated with protease K (1 μg/ml; 10 min at room temperature), fixed in paraformaldehyde (4% for 20 min at 4 °C) and washed in phosphate buffer (pH 7.0). Digoxigenin (DIG)-labelled RNA probes of each of both strands for rat *Prop-1* cDNA were synthesised using DIG RNA labeling Mix (Roche Diagnostics GmbH, Mannheim, Germany) and the AmpliScribe T3 High Yield Transcription Kit (Epicentre, Madison, WI, USA). Hybridisation and colour visualisation were performed according to the manufacturer's manual. Signals of *in situ* hybridisation were present in the cytosol of the cells whose nuclei were stained by immunohistochemistry (Fig. 1c).

### Immunohistochemistry

The embryonic and postnatal pituitaries of Wistar-Imamichi rats and the pituitaries of adult S100β-GFP transgenic rats were fixed with 4% paraformaldehyde in 50 mm phosphate-buffered saline (PBS), pH 7.5, overnight at 4 °C, followed by substitution with 30% sucrose in PBS. Frozen sections of 10 μm thickness in sagittal direction for embryonic day (E)18.5, E19.5 and postnatal day (P)0 pituitaries and in coronal direction for postnatal pituitaries were reacted with primary antibodies at the appropriate dilution at room temperature overnight. Primary antibodies used were guinea pig antiserum against rat PROP1 [dilution 1 : 1000, raised in our laboratory, as described previously (14)], rabbit IgG against cow S-100 (dilution 1 : 100, immunogens are S100β and S100A1; Dako, Ely, UK) and goat IgG against human SOX2 (dilution 1 : 500; Neuromics, Edina, MN, USA). Rabbit antisera against pituitary hormones were: anti-rat αGSU (dilution 1 : 2000), -rat GH (dilution 1 : 8000) and -rat PRL (dilution 1 : 1500), which were provided by the National Institute of Diabetes and Digestive and Kidney Disease (NIDDK) through the courtesy of Dr A. F. Parlow, and anti-human ACTH (dilution 1 : 1000) antibody, which was provided by Dr S. Tanaka at Shizuoka University (Shizuoka, Japan). After washing with PBS, incubation with secondary antibodies was then carried out using fluorescein isothiocyanate- or Cy3-conjugated AffiniPure donkey anti-guinea pig, rabbit and goat IgG (Jackson ImmunoResearch) or Alexa Fluor 488 conjugated goat anti-rabbit IgG (Molecular Probes, Inc., Eugene, OR, USA). The sections were washed with PBS and then enclosed in Vectashield Mounting Medium with 4′,6-diamidino-2-phenylindole (Vector, Burlingame, CA, USA). Immunofluorescence was observed under fluorescence microscopy with a confocal laser scanning microscope (CLSM, Carl Zeiss, Oberkochen, Germany) and BZ-8000 (Keyence, Osaka, Japan).

### Quantitative real-time polymerase chain reaction (PCR)

Total RNAs were extracted from the whole pituitaries at E16.5 (n = 14), E18.5 (n = 11), E20.5 (n = 10), E21.5 (n = 8) and P0 (n = 9), and from the anterior lobes at P5 (n = 4), P10 (n = 8), P15 (n = 8), P30 (n = 6) and P60 (n = 3) using ISOGEN (Nippon Gene, Tokyo, Japan). Reverse transcript was synthesised with Prime Script Reverse Transcriptase (Takara Bio, Kyoto, Japan) using 1 μg of total RNA after DNase 1 treatment and was subjected to quantitative real-time PCR on an ABI Prism 7500 Real-Time PCR System (Applied Biosystems, Foster City, CA, USA). Reaction was performed in SYBR Green-Real Time PCR Master Mix Plus (Toyobo, Osaka, Japan), including 0.6 μm of specific primer set for each gene. Nucleotide sequences of primers used were: *Prop1*, 5′-TCCTGACATCTGGGTTCGAG-3′ and 5′-GGAGTAGTGACCGCTCTTGC-3′; *S100β*, 5′-ACGAGCTCTCTCACTTCCTGGA-3′ and 5′-AGTCACACTCCCCATCCCC-3′; *TAAT box binding protein (Tbp)*, 5′-GATCAAACCCAGAATTGTTCTCC-3′ and 5′-ATGTGGTCTTCCTGAATCCC-3′. Each sample was measured in duplicate in two independent experiments and data were calculated by the comparative C_τ_ method (DDC_τ_ method) to estimate the gene copy number relative to *Tbp* as an internal standard. The DNA sequence of PCR product of each sample was clarified by analysis to be bona fide.

### Statistical analysis

Data from the real-time PCR were analysed using Student's t-test.

## Results

### *Prop1* is expressed in the postnatal anterior lobe

**Fig. 2:**
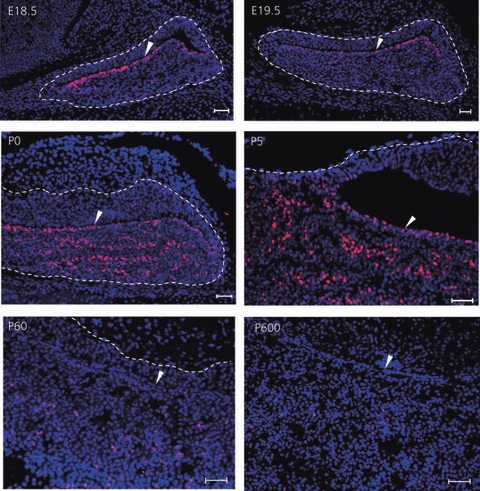
Immunohistochemistry of PROP1. Frozen sections of the embryonic day (E) and postnatal day (P) pituitaries (E18.5, E19.5, P0, P5, P60 and P600) were reacted with anti-PROP1 antiserum. PROP1 is visualised with Cy3 (red) and is overlaid with nuclear staining with 4′,6-diamidino-2-phenylindole (blue). The dotted line and arrowhead indicate the outline of the anterior pituitary and the marginal cell layer, respectively. Scale bar = 50 μm.

We previously reported that immunoreactive signal of PROP1 occupies all of the cells in the primordial pituitary Rathke's pouch at E13.5, then localises in the cells migrating to the expanding anterior lobe at E16.5 (14). In the present study, localisation of *Prop1*-expressing cells was investigated in the rat pituitary from E18.5 to the postnatal period. At E18.5, PROP1-signals were predominantly localised in the marginal cell layer facing the anterior side of the lumen but not in the intermediate side and were scattered in the parenchyma of the anterior lobe (Fig. 2). One day after at E19.5, in addition to lining the marginal cell layer, the stratified pattern of PROP1-signals in the parenchyma of the anterior lobe was observed and became more apparent at P0, followed by decreasing the number of PROP1-positive cells in the marginal cell layer at P5. In the sexually mature pituitary at P60, PROP1-signals were rarely present in the marginal cell layer, although a small number of signals were still observed in the parenchyma of the anterior lobe. This distribution pattern of PROP1-positive cells was mostly maintained at P600 (Fig. 2).

### PROP1-positive cells consistently express *Sox2*

**Fig. 3:**
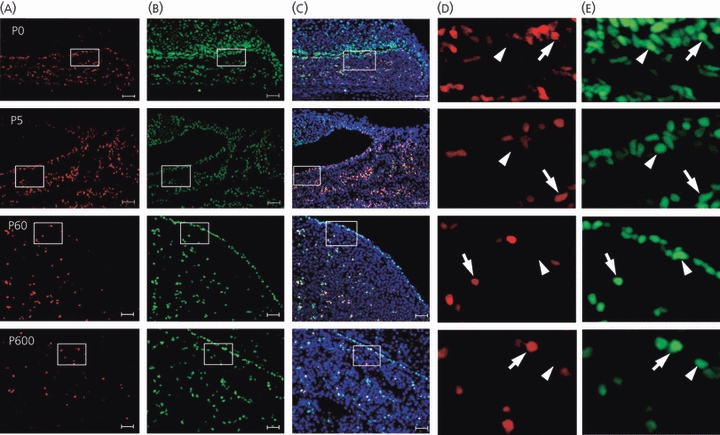
Immunohistochemistry of PROP1 and SOX2. Immunohistochemistry of PROP1 (a) and SOX2 (b) was performed using the rat pituitaries of postnatal day (P)0, P5, P60 and P600. PROP1 (red)- and SOX2 (green)-signals are merged with 4′,6-diamidino-2-phenylindole staining (blue, c) and the boxed area is enlarged (d: PROP1, e: SOX2). (Scale bar = 50 μm). Arrow and arrowhead indicate PROP1/SOX2-positive cell and SOX2-positive cell, respectively.

We previously observed that PROP1-positive cells definitely express *Sox2* in the embryonic pituitaries (14). Double-immunostaining for PROP1 and SOX2 was performed for the postnatal pituitaries of P0, P5, P60 and P600 (Fig. 3). All of the PROP1-positive cells lining the marginal cell layer and stratifying the parenchyma of the anterior lobe at P0 and P5 were positive for SOX2 but not vice versa. At P60 and P600, it is notable that SOX2-positive cells were the major population in the marginal cell layer, where PROP1 signals had almost disappeared, and were scattered in the parenchyma of the anterior lobe where the number of PROP1-positive cells had gradually decreased.

### Significant transition in localisation pattern of PROP1/SOX2-positive cells in the marginal cell layer of the postnatal pituitary

As observed above, the localisation pattern of PROP1-positive cells showed a significant transition. Hence, we performed longitudinal evaluation of the proportion of PROP1-, SOX2- and PROP1/SOX2-positive cells present in the marginal cell layer and the parenchyma of the anterior lobe from P5 to P600 (Table 1). PROP1-positive cells accounted for 68.6% of the cells in the marginal cell layer and 20.8% of the cells in the parenchyma at P5. The values markedly decreased to 5.2% and 7.4%, respectively, at P60, followed by a further decrease to 1.2% and 3.9%, respectively, at P600. Interestingly, the SOX2-positive cells in the marginal cell layer showed a high proportion of 78–90% until P60 and still accounted for 43.8% at P600. On the other hand, the SOX2-positive cells in the parenchyma gradually decreased from 27.3% at P5 to 11.3% at P600.

**Table 1:** Temporal and Spatial Alteration in the Proportion of PROP1- and SOX2-Positive Cells

Marginal cell layer							

		Cell number			Cell population (%)		
			
Postnatal day	n*	DAPI	PROP1	SOX2	PROP1/DAPI	SOX2/DAPI	PROP1 + SOX2/SOX2
5	4	264	181	206	68.6 ± 9.1	78.0 ± 9.1	87.9 ± 3.0
9	4	332	170	NC	51.2 ± 6.6	NC	NC
15	4	286	77	237	26.9 ± 7.1	82.9 ± 2.9	32.5 ± 9.4
60	3	115	6	103	5.2 ± 2.5	89.6 ± 1.8	5.8 ± 2.7
120	6	535	38	286	7.1 ± 2.6	53.5 ± 6.1	13.3 ± 6.2
600	6	489	6	214	1.2 ± 1.2	43.8 ± 10.1	2.8 ± 2.6

Parenchyma							

		Cell number			Cell population (%)		
			
Postnatal day	n*	DAPI	PROP1	SOX2	PROP1/DAPI	SOX2/DAPI	PROP1 + SOX2/SOX2
5	23	2007	418	548	20.8 ± 5.3	27.3 ± 6.3	76.3 ± 10.0
9	22	1832	307	NC	16.8 ± 2.2	NC	NC
15	20	3058	338	536	11.1 ± 3.3	17.5 ± 3.8	63.1 ± 14.6
60	48	3541	262	528	7.4 ± 4.0	14.9 ± 7.3	49.6 ± 25.4
120	44	4391	191	480	4.3 ± 2.6	10.9 ± 3.8	39.8 ± 22.9
600	25	4855	187	549	3.9 ± 2.5	11.3 ± 4.4	34.1 ± 23.9

Cell number of positives was counted for the marginal cell layer and parenchyma of the rat pituitary postnatal day (P)5, P9, P15, P60, P120 and P600. Cell number of PROP1-SOX2 positives was also counted for both areas, and the population of each cell type was calculated.

* n: number of areas (measuring 0.09–0.2 mm^2^) in which 4′,6-diamidino-2-phenylindole (DAPI)-nuclei-stained, PROP1- and SOX2-positives were counted. NC, Not counted.

The proportion of PROP1/SOX2-positive cells in SOX2-positive cells was also determined in the marginal cell layer and the parenchyma (Table 1). Marked decrease in the marginal cell layer was observed from 87.9% at P5 to 5.8% at P60, whereas a modest decrease from 76.3% at P5 to 49.6% at P60 was observed in the parenchyma.

### *Prop1* expressed in non-endocrine cells in the adult anterior lobe

**Fig. 4:**
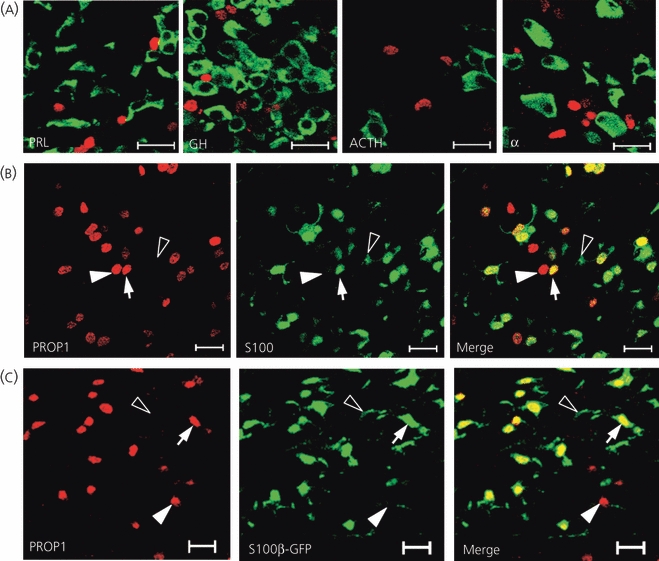
Double-immunohistochemical staining of PROP1 in endocrine- and non-endocrine cells of the adult rat pituitary. Localisation of PROP1 in endocrine cells (a) and non-endocrine cells (b) was examined using the pituitaries of postnatal day (P)60. PROP1 is visualised with Cy3 (red) and pituitary hormones [prolactin (PRL), growth hormone (GH), adrenocorticotrophic hormone (ACTH) and α subunit] and S100 proteins are visualised with fluorescein isothiocyanate (green). Visualisation of S100β was also performed using S100β-green fluorescent protein (GFP) transgenic rat of P60 (c). PROP1 signals localise in S100β-positive non-endocrine cells but not in any endocrine cells. Scale bar = 20 μm. An arrow indicates the PROP1/S100β-positive cell. Open and closed arrowheads indicate the cell positive only for S100 or PROP1, respectively.

We applied double immunohistochemistry using anti-PROP1 and anti-hormone antibodies for the adult rat pituitaries to confirm the previous observation that PROP1 is absent in endocrine cells during the embryonic period (14). As shown in Fig. 4a, none of the endocrine cells coexisted with the PROP1 signal, which is the same as the observations made in the embryonic pituitary, indicating that PROP1-positive cells are non-endocrine cells. Because folliculo-stellate cell is known as a major non-endocrine cell type, immunohistochemistry of S100 protein, which is one of the molecular markers of folliculo-stellate cells, was investigated in the present study. The results obtained demonstrated that *Prop1* was certainly expressed in S100-positive cells (Fig. 4b, arrow). In addition, we found that some cells were positive only for S100 or PROP1 (Fig. 4b, open and closed arrowheads, respectively). For additional confirmation, the immunohistochemistry of PROP1 was examined using the S100β-GFP transgenic rat (22) because S100β-positive cells of the transgenic rat are easily distinguishable by the S100β-promoter-derived expression of the reporter gene, GFP (Fig. 4c). Immunohistochemistry showed the same image as that of a normal rat using anti-S100 antibody (Fig. 4b). Thus, positive cells only for S100β or PROP1 (Fig. 4b, open and closed arrowheads, respectively) were also present.

### Classification of non-endocrine cells in the adult anterior lobe

**Fig. 5:**
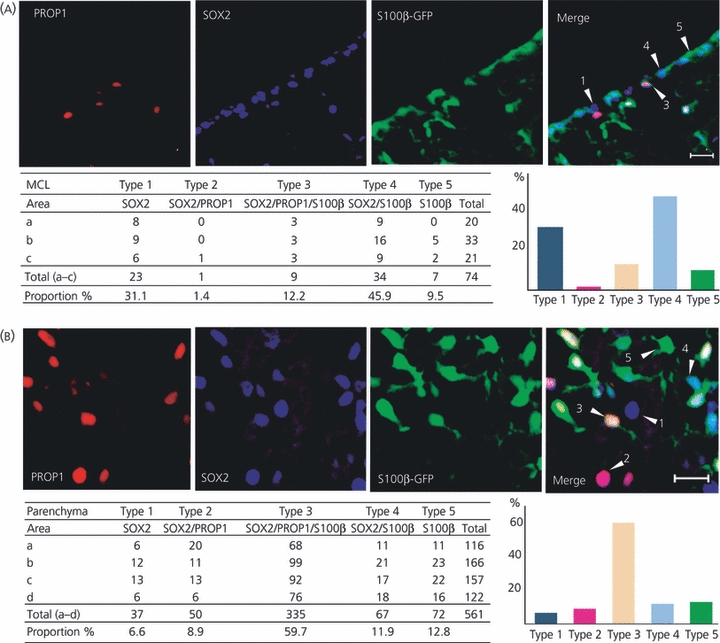
Classification of non-endocrine cells with regard to the expression of *Prop1, Sox2 and S100β.* Immunohistochemistry of adult 60-day-old S100β-green fluorescent protein (GFP) transgenic rat was examined with double immunohistochemical staining of PROP1 (red) and SOX2 (blue) under *S100β*-promoter-derived expression of *GFP* (green). Merged image is shown in the right panel. Scale bar = 20 μm. Expression pattern of *Prop1*, *Sox2* and *S100β* was classified into five cell types (type 1 to type 5). The number of cells belonging to each cell type was counted in randomly selected one area (a, b and c) in each of three sections of two pituitaries for the marginal cell layer (a) and one area (a–d) in each of four sections of two pituitaries for the parenchyma of the anterior lobe (b). The proportion of each cell type was shown as a bar graph.

Classification of non-endocrine cells was examined by localisation of PROP1, SOX2 and S100β using the adult pituitary of S100β-GFP transgenic rats (22). Consequently, we observed five cell populations in the 60-day-old rat pituitary (Fig. 5a): Type1; SOX2-positive, Type2; SOX2/PROP1-positive, Type3; SOX2/PROP1/S100β-positive, Type4; SOX2/S100β-positive, and Type5; S100β-positive.

The number of cells belonging to each cell type in the marginal cell layer and the parenchyma of the anterior lobe of P60 was counted for three and four areas, respectively, and the proportion is shown in Fig. 5. In the marginal cell layer, Type2 (SOX2/PROP1-positive) was only 1.4% and major cell types were Type1 (SOX2-positive, 31.1%) and Type4 (SOX2/S100β-positive, 45.9%) (Fig. 5a). On the other hand, in the parenchyma of the anterior lobe, Type3 (SOX2/PROP1/S100β-positive) was a major population, accounting for 59.7%, whereas the proportions of other cell types ranged from 6.6 to 12.8% (Fig. 5b). It is noteworthy that cells positive only for PROP1 were not present and all of the PROP1-positive cells expressed *Sox2*, together or not with *S100β,* as far as was examined. Classification of non-endocrine cells in the adult anterior lobe demonstrated that the distribution pattern of the SOX2-positive stem/progenitor cell population differs between the marginal cell layer and the parenchyma of the anterior lobe. However, we could not characterise a temporal transition in the distribution pattern because S100β-positive cells were first detected in the postnatal pituitary at P5, when analysed by the immunohistochemical technique (data not shown). Next, we performed quantitative real-time PCR to estimate the mRNA level of the *S100β* in the pituitary of the prenatal to postnatal period.

### Expression profile of *Prop1 and S100β* in pituitary development

**Fig. 6:**
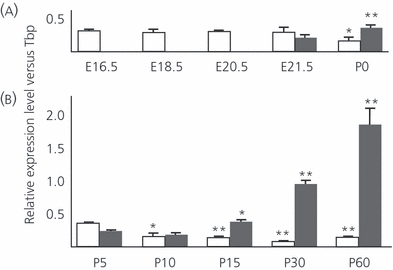
Expression profile of *Prop1* and *S100β* during pituitary development. Quantitative real-time polymerase chain reaction (PCR) was performed to estimate mRNA level of *Prop1* and *S100β* using total RNAs extracted from the whole pituitaries at embryonic day (E)16.5 to postnatal day (P)0 (a) and from the anterior lobes at P5 to P60 (b). Each sample was measured in duplicate in two independent experiments, and data were calculated by comparative C_T_ method to estimate the relative copy number to TAAT box binding protein (Tbp) as an internal standard. The data are presented as the mean ± SD of duplicate PCR in two independent experiments. Open and closed bars indicate *Prop1* and *S100β*, respectively. Significance was examined by comparison with the value of E21.5 (a) or P5 (b). *P < 0.01 and **P < 0.05.

After the late stage of embryonic development, the expression of *Prop1* was specific to the anterior lobe (Fig. 2) and it is known that the expression level of *S100β* is higher in the posterior lobe than that in the anterior lobe (22). For this reason, analysis of the anterior lobe was essential, although it was practically difficult to isolate the anterior lobe from the embryonic pituitary. Therefore, quantitative real-time PCR was performed using total RNAs prepared from the whole pituitaries of prenatal and neonatal periods (Fig. 6a) and from the anterior lobes of the postnatal period (Fig. 6b). The results obtained revealed that *Prop1* was expressed from the prenatal through the postnatal period and its level was gradually decreased. On the other hand, *S100β* started to be expressed at around E21.5 and its expression level increased linearly after P15 in the anterior lobe. Because the expression level of *S100β* was five-fold higher in the posterior-intermediate lobe than in the anterior lobe at P5 (data not shown), the expression level in the anterior lobe at E21.5 and P0 might be low, resulting in a low S100β protein level and failure of detection by the immunoreaction. These data suggest that S100β-positive cells in the anterior lobe first appear at around P0.

## Discussion

In the present study, we aimed to conduct a quantitative and qualitative analysis of pituitary stem/progenitor cells in the postnatal pituitary development by tracking down the cells expressing a stem/progenitor marker *Sox2* and a pituitary-specific transcription factor *Prop1* using immunochemical techniques. Consequently, we demonstrated that a significant transition in the proportion of PROP1/SOX2-positive cells occurred in the marginal cell layer of the rat postnatal pituitary from the neonatal to the adult period, whereas only a gradual quantitative change in the parenchyma of the anterior lobe was observed. Thus, we suggested that a qualitative transition is triggered in the marginal cell layer within the second postnatal week.

Our previous study (14) revealed that the expression of *Sox2* was observed in all of the cells in the invaginating oral ectoderm at E11.5 up to the pituitary primordial Rathke's pouch at E13.5. After E14.5, SOX2-positive cells consistently accounted for almost all of the cells in the marginal cell layer facing both the anterior and intermediate sides of the residual lumen and were also scattered in the expanding parenchyma of the anterior and intermediate lobes. At E16.5, approximately 45% of the cells in the parenchyma of the anterior lobe were SOX2-positive. In the present study, *Sox2* was persistently expressed in the marginal cell layer of both sides during the postnatal period, and 90% of the cells of the marginal cell layer facing the anterior lobe were SOX2-positive even at P60. In the parenchyma of the anterior lobe, the proportion of SOX2-positive cells decreased to 27% at P5 with the progress of pituitary organogenesis and further decreased to 17.5% at P15. During this 2-week postnatal period, the first postnatal growth wave of the rat anterior pituitary occurs as shown by the peak in the number of proliferating cells (24) and observations in the mouse pituitary (13,24,25). Although we did not count the total number of cells, a decrease in the proportion of SOX2-positive cells might be the result of an increased number of cells in the parenchyma of the anterior lobe.

**Fig. 7:**
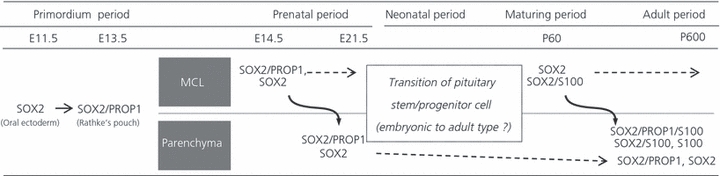
Transition from PROP1 to S100 in stem/progenitor cells during pituitary development. Pituitary stem/progenitor cells express *Sox2* in the early primordium and then start to co-express *Prop1*. During embryonic development of the pituitary, stem/progenitor cells line the marginal cell layer (MCL) and migrate in the parenchyma. After birth, transition of PROP1 to S100 progresses in the stem/progenitor cells at MCL and *Sox2/Prop1/S100*-expressing cells migrate in the parenchyma of the anterior pituitary where *Sox2/Prop1*-expressing embryonic stem/progenitor cells still locate.

On the other hand, the expression of *Prop1* started at E11.5 and eventually accounted for almost all of the cells composing the Rathke's pouch at E13.5. When pituitary organogenesis started to progress, unlike SOX2, PROP1-positive cells existed mainly in the marginal cell layer of the anterior side and in the parenchyma of the expanding anterior lobe; those in the intermediate lobe disappeared completely by the neonatal period (14). After birth, the proportion of PROP1-positive cells on the anterior side of the marginal cell layer decreased gradually from 69% at P5 to 27% at P15, and thereafter rapidly decreased to 5% until P60, whereas the proportion of SOX2-positive cells was maintained at 90% during the same period. Instead of the significant difference between SOX2 and PROP1 in the marginal cell layer, in the parenchyma of the anterior lobe, the proportion of both SOX2- and PROP1-positive cells decreased gradually from 27.3% at P5 to 11.3% at P600 and from 20.8% at P5 to 3.9% at P600, respectively. Characteristically, the PROP1-positive cells consistently expressed SOX2 but not vice versa as far as examined from E11.5 to P600. These data obtained by double-immunohistochemical analysis using anti-PROP1 and anti-SOX2 antibodies confirmed two findings. First, pituitary stem/progenitor cells not only exist mostly in the marginal cell layer, but also in the parenchyma of the anterior lobe. Second, a qualitative transition might be triggered in the marginal cell layer on the anterior side during the first postnatal growth wave of the anterior pituitary, whereas only a gradual quantitative change is observed in the parenchyma of the anterior lobe (Fig. 7).

The marginal cell layer has been proposed to contain a pituitary stem/progenitor cell niche in the postnatal pituitary (10–13,26–30). A tissue stem/progenitor cell is defined by its potency to generate differentiated progenies and its long-term self-renewal capacity. It was shown that *Sox2*-expressing cells are multipotent stem/progenitor cells to generate all of the major cell types in the adult mouse pituitary (12). Analysis of the side population, which is rich in cells with stem/progenitor-like features, revealed that non-Sca1^high^ fraction in the side population of the adult mouse pituitary clusters *Sox2*-expressing cells and possesses an ability to form pituispheres to be differentiated into all of the pituitary endocrine cell types (13). These *Sox2*-expressing stem/progenitor cells are localised in the marginal cell layer but are also scattered in the parenchyma of the anterior pituitary (12,13). Chen *et al.*(13) also reported that SOX2-positive cells are more abundant in the period of the first pituitary growth wave (P7) than in the adult mouse pituitary. These data correspond to our results showing that pituitary *Sox2*-expressing stem/progenitor cells are predominantly localised in the marginal cell layer but are scattered in the parenchyma of the anterior lobe, and also that the number of *Sox2*-expressing cells gradually decreases in the adult anterior lobe. From another point of view in the long-term self-renewal capacity of stem/progenitor cells, a novel technique called telomapping, which visualises the length of telomeres, revealed that cells carrying the longest telomere localise not only mostly in the marginal cell layer, but also in the parenchyma of the anterior lobe. This again indicates the existence of pituitary stem/progenitor cells not only in the marginal cell layer, but also in the parenchyma of the anterior lobe (27). These data correspond to the localisation pattern of *Sox2*-expressing cells described in the present study.

In the present study, we further indicated that PROP1-positive cells consistently express *Sox2* from the prenatal to the postnatal period. By RT-PCR analysis of a side population obtained from the mouse adult pituitary, Chen *et al.*(13) demonstrated that *Prop1* is expressed in the cells belonging to the non-Sca1^high^ fraction that clusters pituitary progenitor cells. Garcia-Lavandeira *et al.* (27)found a pituitary stem/progenitor cell niche characterised by the expression of *Gfra2* (a Ret co-receptor for Neurturin), *Prop1* and stem cell markers such as *Sox2* in the marginal cell layer. However, their immunohistochemical analysis showed that PROP1-positive cells are present only in the marginal cell layer but not in the parenchyma of the rat anterior lobe. Garcia-Lavandeira *et al.* (27) indicated that anti-mouse PROP1antibody gave signals likely in the cytoplasm and in the cytosol/nuclei, in contrast to our data indicating PROP1 signals are observed in the nuclei. In addition, *in situ* hybridisation demonstrated the presence of a *Prop1* signal in the parenchyma of the mouse anterior lobe (15), supporting our data obtained by immunohistochemistry. These discrepancies might be caused by the difference in the properties of anti-PROP1 antibody used. Indeed, the antigens used are somewhat different between the C-terminal region 126–223 of rat PROP1 (present study) and the C-terminal region 151–223 of mouse PROP1 (Garcia-Lavandeira *et al.*). Identity in the C-terminal region 151–223 between mouse and rat is 84%, suggesting a possible difference in antigenicity, as well as in sensitivity.

Recently, Gleiberman *et al.*(26) hypothesised that the adult anterior pituitary is composed of two different terminally-differentiated endocrine cells originating from embryonic precursors and adult stem cells by the cell-lineage labelling technique using Nestin-Cre transgenic mouse. This hypothesis makes it possible to propose that both embryonic precursors and adult stem cells are mixed together in the marginal cell layer and the parenchyma of the anterior lobe from embryonic to postnatal development. Our observation that significant quantitative and qualitative transition in the marginal cell layer occurs during the first postnatal growth wave of the anterior pituitary suggests that a hierarchical change from embryonic precursors to adult stem cells may be triggered in the marginal cell layer during this stage but not in the parenchyma of the anterior lobe. However, Galichet *et al.* (31) presented a dissimilar result using similar Nestin-Cre transgenic mouse. The discrepancy may have arisen from the difference in the gene construct used. Nevertheless, we cannot exclude another possible explanation proposing that there is only one pool of *Sox2*-expressing stem/progenitor cells with a transitioning expression of *Prop1* and *S100β* in the pituitary development. Further investigations are required to clarify this.

In our previous study (14) we proposed that PROP1 exists in *Sox2*-expressing stem/progenitor cells and disappears at the first stage of the transition of progenitor cells to committed cells and does not exist in any endocrine cells during embryonic development. In the present study, we demonstrate that PROP1 mostly exists in S100-positive folliculo-stellate cells but not in any endocrine cells during postnatal development. Although the majority of non-endocrine cells are folliculo-stellate cells, the existence of unidentified cell types in the adult anterior lobe is evident from the accumulating data, such as that obtained from nestin-immunoreactive cells (32). Folliculo-stellate cells themselves remain to be characterised clearly because they show heterogeneity in function, and their origin is unclear (4,5).

In recent years, a number of experimental studies have suggested that folliculo-stellate cells are candidates for pituitary stem/progenitor cells (10,11,33,34). Furthermore, some SOX2-positive stem/progenitor cells express S100 (12,27) and analysis of pituispheres revealed that S100-positive cells appear transiently on the way to generating terminally-differentiated endocrine cells (12). In the present study, we attempted to clarify the correlation among PROP1, SOX2 and S100β using adult S100β-GFP transgenic rats in which folliculo-stellate cells are visualised by GFP expressed under the control of *S100β*-promoter (22). We could classify non-endocrine cells into five groups by the expression pattern of PROP1, SOX2 and S100β: Type1, SOX2-positive; Type2, SOX2/PROP1-positive; Type3, SOX2/PROP1/S100β-positive; Type4, SOX2/S100β-positive; and Type5, S100β-positive. The proportion of each cell type was different in the marginal cell layer and the parenchyma of the anterior lobe. Eighty percent of the non-endocrine cells in the marginal cell layer were Type1 (SOX2-positive, 31%) and Type4 (SOX2/S100β-positive, 46%). On the other hand, the majority of them in the parenchyma of the anterior lobe were Type3 (SOX2/PROP1/S100β-positive, 60%). Because the expression of S100β started at around P0, SOX2/S100β-positive cells would hypothetically be adult stem/progenitor cells. This might mean that SOX2-expressing adult stem/progenitor cells generate SOX2/S100β-positive cells, followed by expressing *Prop1* in the marginal cell layer of the adult pituitary. Then, most of these SOX2/S100β/PROP1-positive cells might migrate into the parenchyma of the anterior lobe and might compose major precursor-endocrine cells in the parenchyma, awaiting the next stimuli to be differentiated. On the other hand, in the parenchyma of the adult anterior lobe, *Sox2*-expressing embryonic stem/progenitor cells are still present to generate SOX2/PROP1-positive cells as precursor-endocrine cells. Thus, endocrine cells in the adult anterior lobe are composed of stem/progenitor cells with a different origin (Fig. 7). From another point of view, PROP1 exists transiently in PIT1-positive commitment cells, although the rate of coexistence declines gradually and reaches almost 0% at E21.5 (14). This coexistence of PROP1 and PIT1 completely disappears in the postnatal anterior lobe (data not shown), suggesting that PROP1 plays different roles or a different developmental system emerges and becomes dominant in the postnatal development. We have postulated the latter possibility, although the transition in the roles of PROP1 remain to be studied. Most recently, it was reported that *prop1* knockdown in zebrafish caused abnormal morphology and gene expression in the early pituitary development, although gradual reverse was observed during late development, suggesting the existence of other fish-specific pathways downstream of *prop1* (35). Although zebrafish is quite a different species from mammals, this result is suggestive for understanding the function of the PROP1 in embryonic and postnatal periods.

In summary, we have demonstrated that pituitary stem/progenitor cells are not only mostly present in the marginal cell layer, but also are scattered in the parenchyma of the anterior lobe. Moreover, a significant quantitative and qualitative transition occurred in the marginal cell layer during the first postnatal growth wave in the anterior pituitary. We propose that this transition might be a hierarchical change in pituitary stem/progenitor cells from those of embryonic to adult stages.

## References

[1] Zhu X, Gleiberman AS, Rosenfeld MG (2007). Molecular physiology of pituitary development: signaling and transcriptional networks. Physiol Rev.

[2] Rinehart JF, Farquhar MG (1953). Electron microscopic studies of the anterior pituitary gland. J Histochem Cytochem.

[3] Vila-Porcile E (1972). The network of the folliculo-stellate cells and the follicles of the adenohypophysis in the rat (pars distalis). Z Zellforsch Mikrosk Anat.

[4] Allaerts W, Vankelecom H (2005). History and perspectives of pituitary folliculo-stellate cell research. Eur J Endocrinol.

[5] Devnath S, Inoue K (2008). An insight to pituitary folliculo-stellate cells. J Neuroendocrinol.

[6] Yoshimura F, Soji T, Kiguchi Y (1977). Relationship between the follicular cells and marginal layer cells of the anterior pituitary. Endocr J.

[7] Yoshimura F, Soji T, Sato S, Yokoyama M (1977). Development and differentiation of rat pituitary follicular cells under normal and some experimental conditions with special reference to an interpretation of renewal cell system. Endocr J.

[8] Correr S, Motta PM (1981). The rat pituitary cleft: a correlated study by scanning and transmission electron microscopy. Cell Tissue Res.

[9] Soji T, Yashiro T, Herbert DC (1989). Granulated ‘marginal cell layer’ in the rat anterior pituitary gland. Tissue Cell.

[10] Vankelecom H (2007). Non-hormonal cell types in the pituitary candidating for stem cell. Semin Cell Dev Biol.

[11] Vankelecom H (2007). Stem cells in the postnatal pituitary?. Neuroendocrinology.

[12] Fauquier T, Rizzoti K, Dattani M, Lovell-Badge R, Robinson IC (2008). SOX2-expressing progenitor cells generate all of the major cell types in the adult mouse pituitary gland. Proc Natl Acad Sci USA.

[13] Chen J, Gremeaux L, Fu Q, Liekens D, Van Laere S, Vankelecom H (2009). Pituitary progenitor cells tracked down by side population dissection. Stem Cells.

[14] Yoshida S, Kato T, Susa T, Cai L-Y, Nakayama M, Kato Y (2009). PROP1 coexists with SOX2 and induces PIT1-commitment cells. Biochem Biophys Res Commun.

[15] Sornson MW, Wu W, Dasen JS, Flynn SE, Norman DJ, O'Connell SM, Gukovsky I, Carriere C, Ryan AK, Miller AP, Zuo L, Gleiberman AS, Andersen B, Beamer WG, Rosenfeld MG (1996). Pituitary lineage determination by the Prophet of Pit-1 homeodomain factor defective in Ames dwarfism. Nature.

[16] Fluck C, Deladoey J, Rutishauser K, Eble A, Marti U, Wu W, Mullis PE (1998). Phenotypic variability in familial combined pituitary hormone deficiency caused by a PROP1 gene mutation resulting in the substitution of ArgCys at codon 120 (R120C). J Clin Endocrinol Metab.

[17] Wu W, Cogan JD, Pfaffle RW, Dasen JS, Frisch H, O'Connell SM, Flynn SE, Brown MR, Mullis PE, Parks JS, Phillips JA, Rosenfeld MG (1998). Mutations in *PROP1* cause familial combined pituitary hormone deficiency. Nat Genet.

[18] Arroyo A, Pernasetti F, Vasilyev VV, Amato P, Yen SS, Mellon PL (2002). A unique case of combined pituitary hormone deficiency caused by a PROP1 gene mutation (R120C) associated with normal height and absent puberty. Clin Endocrinol (Oxf).

[19] Pernasetti F, Toledo SP, Vasilyev VV, Hayashida CY, Cogan JD, Ferrari C, Lourenco DM, Mellon PL (2000). Impaired adrenocorticotropin-adrenal axis in combined pituitary hormone deficiency caused by a two-base pair deletion (301-302delAG) in the prophet of Pit-1 gene. J Clin Endocrinol Metab.

[20] Ward RD, Raetzman LT, Suh H, Stone BM, Nasonkin IO, Camper SA (2004). Role of PROP1 in pituitary gland growth. Mol Endocrinol.

[21] Himes AD, Raetzman LT (2009). Premature differentiation and aberrant movement of pituitary cells lacking both Hes1 and Prop1. Dev Biol.

[22] Itakura E, Odaira K, Yokoyama K, Osuna M, Hara T, Inoue K (2007). Generation of transgenic rats expressing green fluorescent protein in S-100β-producing pituitary folliculo-stellate cells and brain astrocytes. Endocrinology.

[23] Takagi H, Nagashima K, Inoue M, Sakata I, Sakai T (2008). Detailed analysis of formation of chicken pituitary primordium in early embryonic development. Cell Tissue Res.

[24] Taniguchi Y, Yasutaka S, Kominami R, Shinohara H (2001). Proliferation and differentiation of thyrotrophs in the pars distalis of the rat pituitary gland during the fetal and postnatal period. Anat Embryol (Berl).

[25] Chen J, Crabbe A, Van Duppen V, Vankelecom H (2006). The notch signaling system is present in the postnatal pituitary: marked expression and regulatory activity in the newly discovered side population. Mol Endocrinol.

[26] Gleiberman AS, Michurina T, Encinas JM, Roig JL, Krasnov P, Balordi F, Fishell G, Rosenfeld MG, Enikolopov G (2008). Genetic approaches identify adult pituitary stem cells. Proc Natl Acad Sci USA.

[27] Garcia-Lavandeira M, Quereda V, Flores I, Saez C, Diaz-Rodriguez E, Japon MA, Ryan AK, Blasco MA, Dieguez C, Malumbres M, Alvarez CV (2009). A GRFa2/Prop1/stem (GPS) cell niche in the pituitary. PLoS ONE.

[28] Vankelecom H, Gremeaux L (2010). Stem cells in the pituitary gland: a burgeoning field. Gen Comp Endocrinol.

[29] Vankelecom H (2010). Pituitary stem/progenitor cells: embryonic players in the adult gland?. Eur J Neurosci.

[30] Rizzoti K (2010). Adult pituitary progenitors/stem cells: from in vitro characterization to in vivo function. Eur J Neurosci.

[31] Galichet C, Lovell-Badge R, Rizzoti K (2010). Nestin-Cre mice are affected by hypopituitarism, which is not due to significant activity of the transgene in the pituitary gland. PLoS ONE.

[32] Krylyshkina O, Chen J, Mebis L, Denef C, Vankelecom H (2005). Nestin-immunoreactive cells in rat pituitary are neither hormonal nor typical folliculo-stellate cells. Endocrinology.

[33] Inoue K, Taniguchi Y, Kurosumi K (1987). Differentiation of striated muscle fibers in pituitary gland grafts transplanted beneath the kidney capsule. Arch Histol Jpn.

[34] Mogi C, Miyai S, Nishimura Y, Fukuro H, Yokoyama K, Takaki A, Inoue K (2004). Differentiation of skeletal muscle from pituitary folliculo-stellate cells and endocrine progenitor cells. Exp Cell Res.

[35] Angotzi AR, Mungpakdee S, Stefansson S, Male R, Chourrout D (2011). Involvement of Prop1 homeobox gene in the early development of fish pituitary gland. Gen Comp Endocrinol.

